# Correction to “Sleep disturbance in alopecia areata: A cross‐sectional study”

**DOI:** 10.1002/hsr2.1973

**Published:** 2024-03-11

Shakoei S, Torabimirzaee A, Saffarian Z, Abedini R. Sleep disturbance in alopecia areata: a cross‐sectional study. *Health Sci Rep*. 2022;e576. doi:10.1002/hsr2.576

It has come to our attention that the second affiliation of Alireza Torabimirzaee was not mentioned.

The corrected affiliations appear as follows:

Safoura Shakoei^1^ | Alireza Torabimirzaee^1,3^ | Zahra Saffarian^1^ | Robabeh Abedini^2^

^1^Department of Dermatology, Imam Khomeini Hospital, Tehran University of Medical Sciences (TUMS), Tehran, Iran

^2^Department of Dermatology, Razi Hospital, Tehran University of Medical Sciences (TUMS), Tehran, Iran

^3^Department of Radiology, School of Medicine, Iran University of Medical Sciences, Tehran, Iran

We apologize for this error.

